# An Improved Multimodal Biometric Identification System Employing Score-Level Fuzzification of Finger Texture and Finger Vein Biometrics

**DOI:** 10.3390/s23249706

**Published:** 2023-12-08

**Authors:** Syed Aqeel Haider, Shahzad Ashraf, Raja Masood Larik, Nusrat Husain, Hafiz Abdul Muqeet, Usman Humayun, Ashraf Yahya, Zeeshan Ahmad Arfeen, Muhammad Farhan Khan

**Affiliations:** 1 Department of Computer & Information Systems Engineering, Faculty of Computer & Electrical Engineering, N.E.D. University of Engineering and Technology, Karachi 75270, Pakistan; 2 Department of Electrical Engineering, NFC Institute of Engineering and Technology, Multan 60000, Pakistan; nfc.iet@hotmail.com; 3 Department of Electrical Engineering, N.E.D University of Engineering and Technology, Karachi 75270, Pakistan; rmlarik@neduet.edu.pk; 4 Department of Electronics & Power Engineering, Pakistan Navy Engineering College, National University of Sciences and Technology (NUST), Islamabad 44000, Pakistan; nusrat@pnec.nust.edu.pk (N.H.); ayahya@pnec.nust.edu.pk (A.Y.); farhankhan@pnec.nust.edu.pk (M.F.K.); 5 Electrical Engineering Technology Department, Punjab Tianjin University of Technology, Lahore 54770, Pakistan; abdul.muqeet@ptut.edu.pk; 6 Department of Computer Engineering, Faculty of Engineering, Bahauddin Zakariya University (BZU), Multan 60800, Pakistan; usmanhumayun@bzu.edu.pk; 7 Department of Electrical Engineering, The Islamia University of Bahawalpur, Bahawalpur 63100, Pakistan; zeeshan.arfeen@iub.edu.pk

**Keywords:** biometric modalities, convolutional neural network, Finger Texture biometric, Finger Vein biometric, Fuzzy Inference System, Linear Binary Pattern, multimodal biometric system, Support Vector Machine

## Abstract

This research work focuses on a Near-Infra-Red (NIR) finger-images-based multimodal biometric system based on Finger Texture and Finger Vein biometrics. The individual results of the biometric characteristics are fused using a fuzzy system, and the final identification result is achieved. Experiments are performed for three different databases, i.e., the Near-Infra-Red Hand Images (NIRHI), Hong Kong Polytechnic University (HKPU) and University of Twente Finger Vein Pattern (UTFVP) databases. First, the Finger Texture biometric employs an efficient texture feature extracting algorithm, i.e., Linear Binary Pattern. Then, the classification is performed using Support Vector Machine, a proven machine learning classification algorithm. Second, the transfer learning of pre-trained convolutional neural networks (CNNs) is performed for the Finger Vein biometric, employing two approaches. The three selected CNNs are AlexNet, VGG16 and VGG19. In Approach 1, before feeding the images for the training of the CNN, the necessary preprocessing of NIR images is performed. In Approach 2, before the pre-processing step, image intensity optimization is also employed to regularize the image intensity. NIRHI outperforms HKPU and UTFVP for both of the modalities of focus, in a unimodal setup as well as in a multimodal one. The proposed multimodal biometric system demonstrates a better overall identification accuracy of 99.62% in comparison with 99.51% and 99.50% reported in the recent state-of-the-art systems.

## 1. Introduction

Automated biometric identification based on anatomical human characteristics is widely available in commercial products today. These include banking, immigration, consumer electronics, e-governance and e-commerce applications.

The human characteristic may be declared as a biometric modality if it has certain qualities. The main qualities are universality, distinctiveness, permanence, collectability, performance, acceptability and circumvention [1]. All these qualities must be present to some extent in a biometric human characteristic.

- Universality means that all humans must have that characteristic in them.
- Distinctiveness means, for each individual, the characteristic must be present in some different form.
- Permanence shows that the biometric characteristic must remain invariable over a sufficient period.
- Collectability means the biometric characteristic must be quantifiable, such that it may be collected from human subjects.
- Performance shows the ability to identify human subjects on the basis of that characteristic.
- The degree of ease and obstructiveness defines the degree of the acceptability of the biometric modality.
- Circumvention describes the degree of difficulty required to launch a spoof attack to deceive the biometric-modality-based system.

Human characteristics are divided into two main types: physiological and behavioral. Physiological characteristics are related to the human body, whereas behavioral characteristics belong to human routine actions. Physiological characteristics mostly remain constant, whereas behavioral characteristics are more vulnerable to change over time [1]. Physiological characteristics are further divided into extrinsic and intrinsic modalities. Extrinsic physiological characteristics are found outside the human body, whereas intrinsic modalities are present inside. Extrinsic modalities are open and comparatively easier to deceive using planned imposter attacks. Finger Veins are categorized under the intrinsic type of physiological human characteristic. There are some obvious reasons for working with Finger Vein biometrics. 

First, the use of the vein structure of the hand or finger as a biometric trait is preferred because of the high degree of privacy. Vein patterns are hidden underneath the skin and are almost impossible to capture under visible lighting conditions. Hence, this characteristic enormously increases the reliability of a biometric system against spoof attacks. The second advantage of using the vein structure of the hand or finger is that it improves the integrity and lifetime of the biometric system. The reason behind this is the high difficulty level of altering the vein pattern within the human body through some surgical procedures [2]. The third reason for the widespread use of Finger Veins as a biometric trait is their acceptability. Finger Vein images may be captured under noncontact or weak-contact conditions. Therefore, the biometric data acquisition setup becomes more user-friendly. The lower vulnerability of Finger Vein data collection to scars, sweat or the presence of some unwanted material on the finger surface makes it more interference-resistant. Because of this quality, Finger Vein biometrics are preferably used in biometric systems.

In the past two decades, many physical and behavioral biometric modalities have been under extensive research, such as fingerprints, palm prints, palms/Finger Textures, faces, irises, voice, gait and signature [1]. All these modalities are vulnerable to presentation spoof attacks; hence, the level of provided security is compromised. Fingerprint- and palm-print-based biometric systems may be deceived by using gelatin or clay-made artificial fingerprint surfaces or images. Biometric systems using faces as a biometric modality may be attacked by using photographs, 3-D face models and recorded short clips. Iris-based biometric systems may encounter spoof attacks by employing iris images taken from enrolled users. Voice- and gait-based biometric systems may be attacked by feeding prerecorded audio and video to the recognition system, respectively. In the same way, signatures of enrolled users may be acquired to attack the biometric system by some unauthorized spoof. In the case of a Finger-Vein-based biometric system, because of the intrinsic nature of the biometric data, launching a spoof attack is itself a hectic and nearly impossible task.

**Figure 1:**
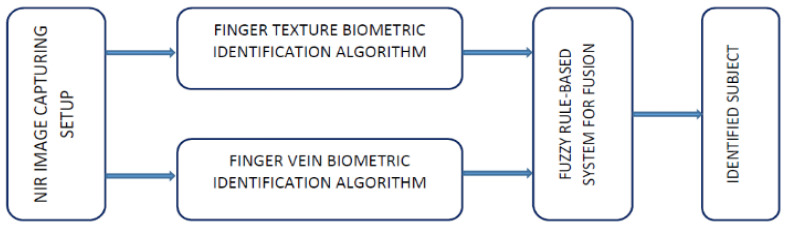
Block diagram for proposed multimodal biometric system.

We considered two biometric modalities for our proposed multimodal biometric system: the Finger Texture biometric and the Finger Vein biometric. Both biometrics are treated as intrinsic biometrics because we used finger images captured with an NIR filter-mounted camera. The NIR camera captures the vein patterns and textures hidden behind human skin. The overall block diagram for the proposed system is shown in Figure 1.

The main contributions are as mentioned below:

- We employed a method of intensity optimization for the Near-Infra-Red Finger Vein images extracted from the NIRHI database. The experimental results show that intensity optimization improves the overall identification performance of the biometric system.
- We proposed an algorithm for processing the databases (NIR Hand Images database and acquired databases) to extract texture features and generate unimodal identification results based on the performed experiments.
- The proposed NIR Hand Images database and two on-request acquired databases were processed through three recently proposed pre-trained convolutional neural networks (AlexNet, VGG16 and VGG19) for having the identification accuracy parameters. The experimental results clearly show better performance accuracy for the unimodal biometric system.
- The proposed Fuzzy Rule-Based Inference system enhances the overall identification accuracy of the multimodal biometric system in comparison with individual identification accuracies.

This research article is structured as follows: Related Works, Methodology, Experimental Setup and Results, Simulation and Discussion, Comparative Analysis and Conclusions.

## 2. Related Works

According to the latest research, Finger-Vein-biometric-based systems may also have spoof attacks [1,2,3]. For real applications, a biometric system’s excellent capability against spoof attacks is required. Many researchers have proposed presentation attack detection (PAD) techniques for different biometric modalities. Researchers have proposed PAD techniques for Finger Vein biometrics in [3,4,5,6,7,8]. The lack of standardization, compromising the user comfort factor and not following ethical implications are shortcomings of these research works.

Over time, many techniques have been deployed to process Finger Vein images for the generation of recognition results. The researchers of [9,10,11,12,13,14,15] have presented various techniques to improve the quality of captured Finger Vein images.

S. A. Haider et al. [3] proposed a multimodal biometric system based on intrinsic biometric modalities. They employed Finger Veins, Hand Geometry and Pulse Response as the modalities of focus. Pulse Response biometrics were employed to filter illegitimate candidates by filtering out spoof attacks launched using artificially made non-living human hands. The Near-Infra-Red Hand Images database was maintained and processed for Hand Geometry and Finger Vein biometrics separately. Matching scores were then fused at the score level using a Fuzzy Inference System to produce a final decision regarding the identification of the candidate subject.

R. Das et al. [16] proposed a convolutional-neural-network-based Finger Vein identification system. They evaluated the performance of their proposed system using four publicly available databases. They claimed to achieve accuracy beyond 95% for all four employed databases. The claimed accuracy needs to be improved further. Comparisons for no other performance metrics were reported other than accuracy. The authors of [17] proposed a multimodal biometric system using Finger Vein and fingerprint biometrics. They used feature-level fusion and reported using a new NIR imaging device to capture images. The overall recognition rate for the proposed system was claimed as 96.93% with a 0% False Acceptance Rate. The recognition rate had a margin for improvement, and the False Rejection Rate was not discussed in the conclusions.

Researchers of NIR [18] explored remote photo-plethysmography to identify and prevent illegitimate user attacks using artificially generated Finger Vein data. An overall accuracy of 96.46% was reported which may be improved further. Although more comparisons may be included in terms of performance metrics. The algorithm proposed in another research article [19] claimed to present a novel and effective technique regarding region of interest localization for the Finger Vein biometric system. They discussed only the Equal Error Rate and showed a comparison, but an overall accuracy discussion was missing. In [20], an efficient multimodal biometric system was proposed using an image-capturing setup based on Finger Vein and finger shape biometric modalities. They recovered the shapes of fingers by employing their proposed method. The finger shape part of their research work may easily be broken into using a fake finger imposter attack. In [21], the researchers discussed iris-, face- and Finger-Vein-biometric-based multimodal biometric systems employing deep learning techniques. This method is less user-friendly, as three different parts of the body are involved in biometric data capturing. The claimed accuracy values are unrealistically high, i.e., 100% and 99.39% for score-level and feature-level fusion, respectively. The researchers of [22] proposed an efficient method for the enhancement of NIR Finger Vein images. The proposed method is only for enhancing NIR images, but a discussion regarding the overall recognition system was not included in the research article.

In [23], supervised discrete hashing and convolutional neural networks were employed for a Finger-Vein-based biometric authentication system. The researchers reported unmatched performance after comparison with a publicly available two-session Finger Vein database. The researchers of [24] presented a technique of employing fuzzy inference system to propose Multi-criteria decision-making method. This proposed method is used for prioritizing the health and safety risks that may occur during an experiment. 

The authors of [25] also employed a convolutional neural network for Finger Vein biometric identification. They reported performing a comparative analysis of different databases and also considered environmental changes. They maintained new image databases and reported that, after testing, performance was improved in comparison with existing systems. In [26], an accurate and reliable multimodal biometric system was proposed using fingerprint, Finger Vein and face biometrics. The publicly available SDUMLA-HMT database was used for testing and evaluation purposes. They generated matching scores for the three modalities of focus and implemented score-level fusion. The recognized subject was declared after comparing the overall score with a predefined threshold. 

The researchers of [27] proposed an efficient noise removal algorithm without affecting the texture features present in the Finger Vein images. They claimed to preserve texture features using the proposed denoising algorithm, which was tested on the training database. Different types of noises like Poisson, salt-and-pepper, Gaussian and Speckle noise were introduced into the training database for a performance evaluation of the proposed algorithm in comparison with traditional and famous denoising algorithms. They reported improved performance for the proposed algorithm. 

In another article [28], the researchers discussed a multimodal biometric identification system considering Finger Veins and palm veins as biometric traits. They performed preprocessing of the captured vein images, employing a revised 2-D Gabor filter and a gradient-based technique, before the extraction of features for the modalities of focus. The extracted features were matched using the Euclidean Distance metric. The matching scores were then fused at the score level using a Fuzzy Inference System. They reported performing experiments on standard databases for Finger Vein and palm vein images. They claimed improved performance parameters for the biometric system.

The authors of [29] proposed a multimodal biometric system consisting of three biometric modalities: irises, palm veins and Finger Veins. They reported employing a hybrid fusion model comprising an enhanced feature fusion algorithm and a novel weighted voting strategy. They tested the proposed system on databases from CASIA, PolyU and SDU and reported improved recognition accuracy and reliability in comparison with existing multimodal systems. They claimed to achieve an average recognition accuracy of 99.33%. The level of acceptability and the degree of ease of use were low for this research, as two body parts were involved in biometric raw data collection. The researchers of [30] proposed new architecture for a CNN. They named it Xception. They tested it on two different databases, i.e., the SDUMLA and THU-FVFDT2 datasets. They reported improved performance metrics. Table 1 summarizes the cited multimodal biometric systems.

The research findings for the discussed research articles [23,25,26,27,28,29] are lacking in terms of ease of the methodology followed. There is still space for better overall accuracy and other evaluation metrics like False Acceptance Rate, False Rejection Rate, etc. Further, the overall training and testing times for the proposed methods may be improved. Resistance capabilities against any imposter attack are lesser wherever extrinsic modalities are employed in the proposed biometric systems.

## 3. Methodology

The methodology for individual biometric modalities (Finger Textures and Finger Veins) is explained in the following section.

### 3.1. Image Databases

In this work, the NIR Hand Images database was used [3]. To perform a comparative analysis, we acquired two publicly available databases, the HKPU Finger Vein images database [31] and the UTFVP database [32,33], collected from Hong Kong Polytechnic University and the University of Twente Netherlands, respectively. Figure 1 shows a block diagram of the proposed system.

**Figure 2:**
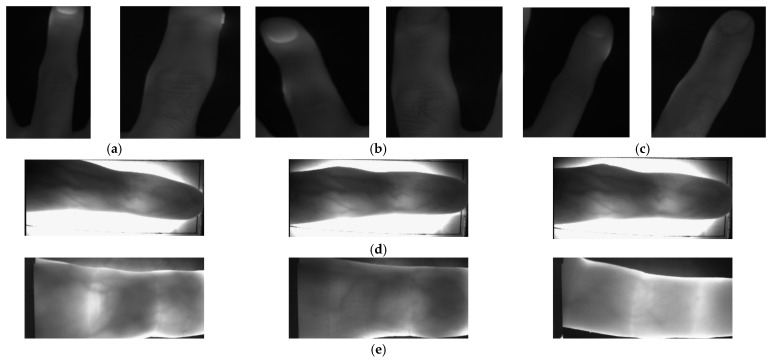
Sample images for NIRHI, HKPU and UTFVP databases. (**a**) Center finger—NIRHI sample images. (**b**) Ring finger—NIRHI sample images. (**c**) Index finger—NIRHI sample images. (**d**) HKPU sample images. (**e**) UTFVP sample images.

The individual identification results (Finger Texture and Finger Vein biometrics) were fused using a Fuzzy Inference System to generate the final identification result, which is depicted in Figure 2.

#### 3.1.1. Near-Infra-Red Hand Images Database (NIRHI)

The Manta G145B—GigE—Camera of Allied Vision was used for the acquisition of the NIRHI database. It is a 1.4-megapixel monochrome camera. The resolution of each captured image is 1360 × 1024 pixels. A near-infrared (NIR) filter was installed on the aperture of the camera to capture NIR images. This camera was mounted on a metallic alloy stand facing downward. A lighting pad was specially designed as a lighting source. The lighting pad had high-intensity LEDs installed on it, and it was installed on the floor of the metallic alloy stand. The intensity of the lighting source and the distance between the camera and the lighting pad were adjusted at the start of the database acquisition process. These arrangements remained constant for the duration of the image-capturing sessions to maintain the uniformity of the database images. The images were captured from 185 subjects with 200 images per subject, comprising undergraduate and postgraduate students, as well as some university employees [3]. This database was preprocessed further to have the desired finger images fed into classification algorithms. Two sample images per finger, i.e., the center, ring and index fingers, are shown in the above-mentioned Figure 2a–c.

#### 3.1.2. Hong Kong Polytechnic University Database (HKPU)

In this acquired database [31], images for 156 subjects are available. For subject numbers 1–105, there are 24 images per subject, whereas for subject numbers 106–156, there are 12 images per subject, which were duplicated to obtain 24 images per subject. This database was preprocessed further to have the desired images fed into our classification algorithms. Three sample images for the fingers are shown in Figure 2d.

#### 3.1.3. University of Twente Finger Vein Pattern (UTFVP)

In this acquired database [32,33], we found images for 110 subjects. For each subject, there are 24 images per subject. This database was preprocessed further to have the desired images fed into our classification algorithms. Three sample images for the fingers are included in Figure 2e for this database.

**Figure 3:**
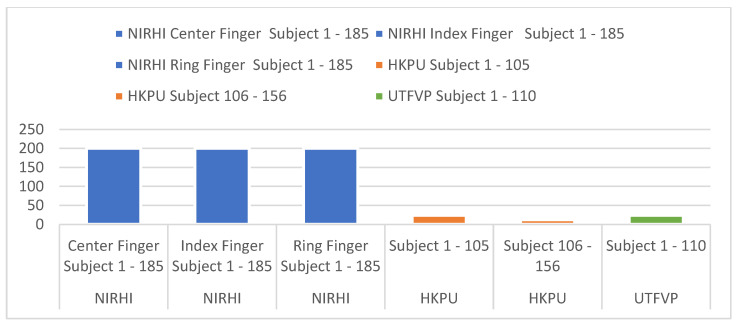
Details of images in the employed databases.

The details regarding the number of images in the NIRHI database and two acquired databases, i.e., HKPU and UTFVP, are mentioned in Figure 3.

### 3.2. Finger Texture Biometric

**Figure 4:**
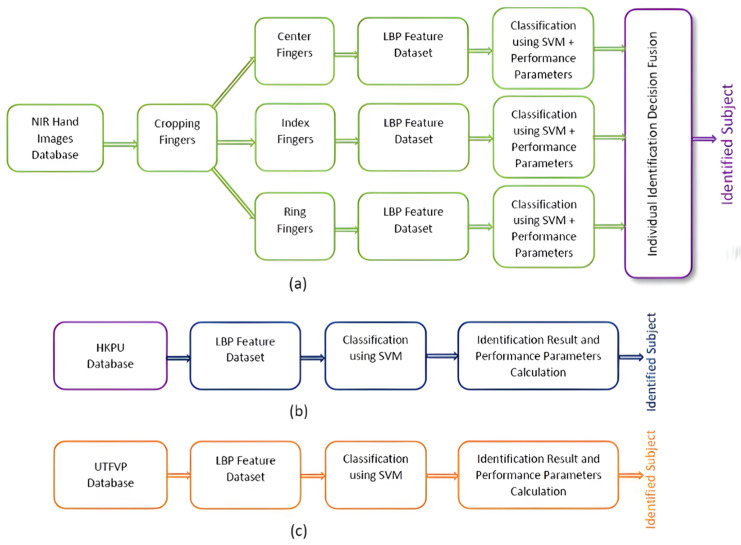
Finger Texture identification algorithm for (**a**) NIRHI, (**b**) HKPU and (**c**) UTFVP.

The step-by-step algorithm for the Finger Texture biometric is explained below. Figure 4 displays the schematic diagram for the identification algorithm.

#### 3.2.1. Preprocessing

The images of the Near-Infra-Red Hand Images database [3] were cropped to have images for the index, middle and ring fingers. In this way, we had 600 finger images per subject (200 images per finger). These finger images were to be fed into the classification algorithm.

In the acquired databases [31,32,33], we already had Finger Vein images. Therefore, no further preprocessing was required before feeding the images into the Finger Texture classification algorithm.

#### 3.2.2. Linear Binary Pattern

We employed the Linear Binary Pattern algorithm to extract texture features from the NIR finger images. For all three image databases of concern, we obtained a 1 × 10 feature matrix per image.

#### 3.2.3. Support Vector Machine

Support Vector Machine was selected as the classification algorithm after a comparative analysis with other machine learning classification algorithms. The model for multiple-subject Support Vector Machine was trained and tested to obtain the accuracy and other performance parameters. First, a sample of a 1 × 10 LBP feature matrix was selected to be used for classification using Support Vector Machine. Different sample sizes were tried and reported for all three image databases of concern, as discussed in the Experimental Results section. 

After the completion of the training process, the trained model was tested using the testing samples to evaluate the accuracy and other performance parameters.

### 3.3. Finger Vein Biometric

**Figure 5:**
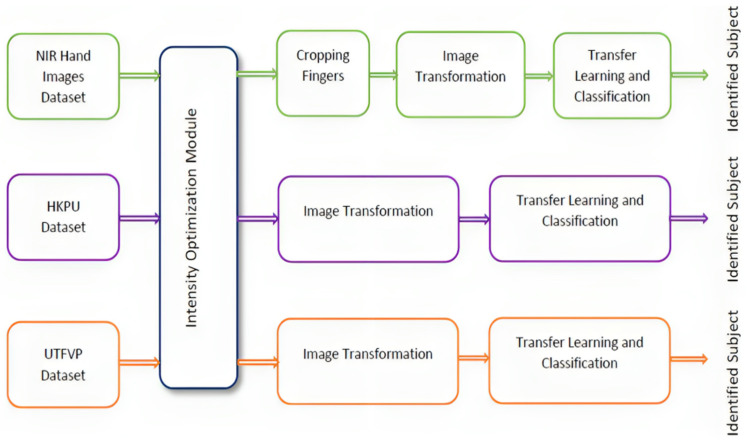
Finger Vein identification algorithm for NIRHI, HKPU and UTFVP Databases.

The steps for the Finger Vein biometric algorithm are shown in Figure 5. Each step is explained in the discussion below.

#### 3.3.1. Image Intensity Optimization

**Figure 6:**
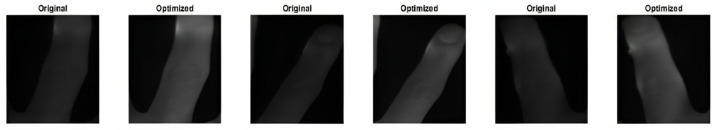
Original and optimized images.

In the first step, NIR finger images were processed to optimize of the intensity level. To achieve a comparable accuracy and optimal performance for the classification algorithm, it is required to have a similar level of pixel intensity throughout the database images. Some of the original and processed images are shown in Figure 6.

**Figure 7:**
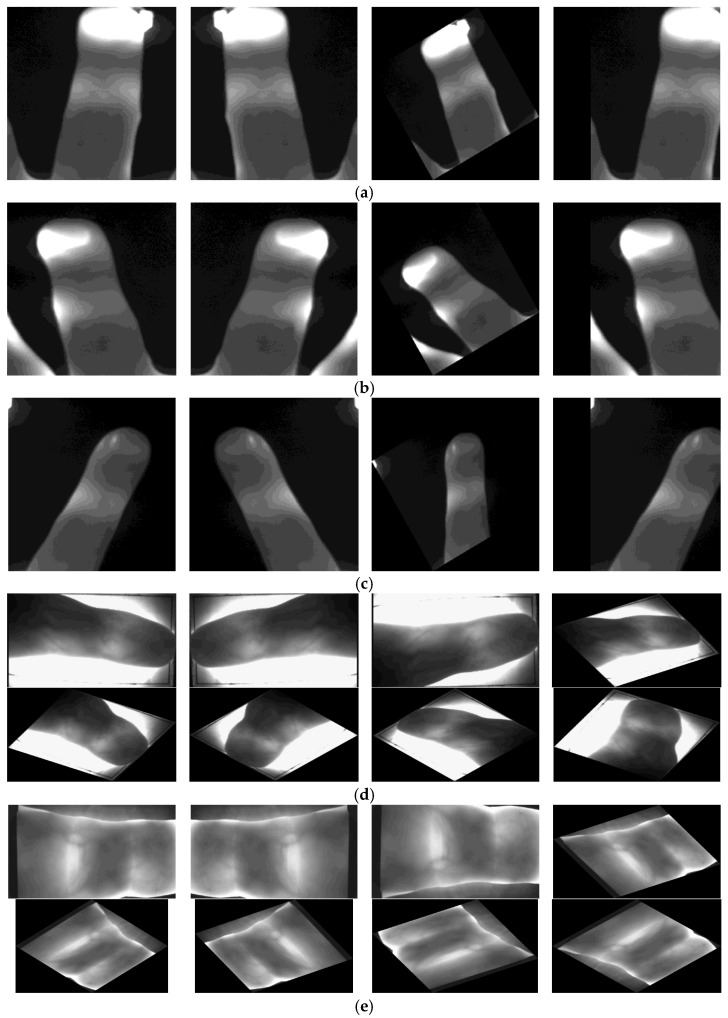
Sample transformed images for the three databases. (**a**) NIRHI—center finger images after transformation. (**b**) NIRHI—index finger images after transformation. (**c**) NIRHI—ring finger images after transformation. (**d**) HKPU—sample finger images after transformation. (**e**) UTFVP—sample finger images after transformation.

The optimization is achieved by examining the overall intensity level of image pixels and, depending upon it, increasing or decreasing the intensity of each pixel by employing intensity scaling. Therefore, all three employed databases [3,31,32,33] were preprocessed through coding in MATLAB to achieve smoothened and intensity-optimized finger images, as displayed in Figure 7.

#### 3.3.2. Image Transformation

The proposed NIR Finger Vein database was preprocessed before feeding it into AlexNet to generate identification accuracy results using transfer learning. The images were cropped to have separate images for the index, center and ring fingers. Therefore, after this step, there were 600 images per subject in our database. These images were further transformed to enhance the number of images to be fed into the classification algorithm by shifting them 50 pixels along the *x*-axis, flipping them and rotating them by 30°. Hence, after transformation, we had 2400 images per subject in our proposed database.

The HKPU and UTFVP databases were also preprocessed before feeding the images into AlexNet. There were 156 and 110 subjects in the HKPU and UTFVP databases, respectively. Initially, per subject, the number of images was 24 and 12 for the HKPU and UTFVP databases, respectively. UTFVP images were duplicated to have 24 images per subject. The images were transformed to increase the number of images per subject. First, each image was resized to 224 × 224 pixels. Then, the resized image was shifted along the *x*-axis and *y*-axis by 50 pixels. Further, the resized image was flipped horizontally and vertically. After that, the resized image, along with the two flipped images, was rotated clockwise and anti-clockwise 30°, 45° and 60°. The number of images per subject became 552 for both databases. 

The sample transformed images are shown in Figure 7 for all three databases.

This image transformations/augmentations help in avoiding the overfitting problem for convolutional neural networks.

#### 3.3.3. Size Conversion and Transfer Learning

We employed transfer learning using pre-trained AlexNet [34], VGG16 [35] and VGG19 [36] to obtain classification results for Finger Vein images. Therefore, we converted the databases into Finger Vein images of desired shape specifications. AlexNet requires RGB images as input with an image size of 227 × 227, whereas VGG16 and VGG19 require RGB images with an image size of 224 × 224 as input. Therefore, before feeding the transformed databases into the pre-trained model of the convolutional neural network for transfer learning, the images were resized according to the respective requirements during the image transformation step. Transfer learning was implemented by changing the last fully connected layer and classification output layers according to our requirements. For AlexNet, layer numbers 23 and 25 were altered. For VGG16, layers 39 and 41 were altered. For VGG19, layers 142 and 144 were altered.

#### 3.3.4. Architecture of Convolutional Neural Networks

AlexNet has eight layers; the first five are convolutional layers. Out of the five, three convolutional layers (1, 2 and 5) are followed by max pooling layers. Then, there are three fully connected layers along with drop-out layers, except the last fully connected layer. All convolutional and fully connected layers have a ReLU activation function, except the last fully connected layer. The last fully connected layer, which is of the linear type, is followed by the softmax output layer.

VGG16 has five sets of convolutional layers followed by max pooling layers. In the first two sets, there are two convolutional layers, whereas the remaining sets have three convolutional layers. All the convolutional and fully connected layers have a ReLU activation function. In the end, there are three fully connected layers before a softmax layer as the output layer.

VGG19 also has five sets of convolutional layers followed by max pooling layers. In the first two sets, there are two convolutional layers, whereas the remaining sets have four convolutional layers. All the convolutional and fully connected layers have a ReLU activation function. In the end, there are three fully connected layers followed by a softmax layer for the output.

## 4. Experimental Setup and Results

The experiments essentially employed MATLAB coding for both biometric modalities of concern, i.e., Finger Textures and Finger Veins. The experimental steps and observed performance parameters for each of the modalities are discussed separately in the subsections. After the calculation of the confusion matrix parameters, as shown in Table 2, i.e., True Positive (TP), True Negative (TN), False Positive (FP) and False Negative (FN), the performance parameters, i.e., Accuracy, Precision, Recall (True Positive Rate—TPR), F1 Score, True Negative Rate (TNR), False Rejection Rate (FRR)/False Positive Rate (FPR) and False Acceptance Rate (FAR)/False Negative Rate (FNR), were calculated as evaluation metrics for our models. The formulas for these parameters are shown in Equations (1)–(7):

(1) $$\mathrm{Accuracy}=\frac{\left(\mathrm{TP}+\mathrm{TN}\right)}{\left(\mathrm{TP}+\mathrm{TN}+\mathrm{FP}+\mathrm{FN}\right)}$$

(2) $$\mathrm{Precision}=\frac{\mathrm{TP}}{\mathrm{FP}+\mathrm{TP}}$$

(3) $$\mathrm{Recall}\;\left(\mathrm{TPR}\right)=\frac{\mathrm{TP}}{\mathrm{FN}+\mathrm{TP}}$$

(4) $$F1\;\mathrm{Score}=2\times \frac{\mathrm{Precision}\;\times \;\mathrm{Recall}}{\mathrm{Precision}+\mathrm{Recall}}$$

(5) $$\mathrm{TNR}=\frac{\mathrm{TN}}{\mathrm{TN}+\mathrm{FP}}$$

(6) $$\mathrm{FRR}=\;\mathrm{FPR}=\frac{\mathrm{FP}}{\mathrm{FP}+\mathrm{TN}}$$

(7) $$\mathrm{FAR}=\;\mathrm{FNR}=\frac{\mathrm{FN}}{\mathrm{FN}+\mathrm{TP}}$$

### 4.1. Finger Texture Biometric

Each of the databases of concern was converted into texture feature matrix databases using Linear Binary Pattern. Initially, the sample from the feature matrix database was divided into training and testing samples with a ratio of 80:20. The five best features were selected out of ten to train the model with the feature matrix training samples. Five-way cross-validation was employed while training the model using the best features to avoid the model overfitting problem. 

Overfitting means the model accuracy of classification is excellent if it is tested for the images on which it was trained. But, when the model is tested using images that are not taken from the training set, its accuracy of classification drops significantly. In terms of epochs, if the number of epochs increases, the training accuracy increases, whereas the test and validation accuracies decrease. This is called overfitting.

The Statistics and Machine Learning Toolbox of MATLAB 2021a was employed. From the library, the function ‘sequentialfs’ was used for the sequential forward feature selection of the 5 best-performing parameters out of 10 employing cross-validation. The main hyperparameters for SVM models are Box Constraint and Kernel Scale, which may be tuned to build a better classification model. Auto optimization of hyperparameters was selected during SVM model training using the ‘fitcecoc’ MATLAB function. ‘Expected improvement plus’ was used as the ‘acquisition function’ under the ‘hyperparameter optimization options’. In addition to this, ‘show plots’ was set as true.

As there were three databases of NIR finger images with different numbers of subjects and numbers of images per subject, each one was dealt with separately, as explained below.

#### 4.1.1. NIRHI Database

There was a total of 185 subjects and 200 NIR images per subject for the center, index and ring fingers after the preprocessing step. These finger images were processed to obtain three separate databases of texture feature matrices for the center, index and ring finger images. In each of the LBP texture feature matrix databases, there were 200 matrices for each one of the 185 subjects. We selected a random sample of feature matrices out of 200. We maintained sample databases of 30, 25 and 20 feature matrices per subject. These sample databases were employed for classification using SVM model coding. The classification accuracies and elapsed time are presented in Table 3.

#### 4.1.2. HKPU Database

There were a total of 24 NIR finger images for each of the 156 subjects in this acquired database. These images were converted into LBP texture feature matrices, and the database was maintained. This feature matrix database had 24 matrices per 156 subjects. The selected sample databases of 24, 22 and 20 feature matrices per subject were used to train and test the SVM classification model. The response of the SVM classification model is presented in Table 4 in the form of classification accuracies and consumed time.

#### 4.1.3. UTFVP Database

For this database, there was a lesser number of images per subject, i.e., 12 images for each of the 110 subjects. The same procedure was repeated for this database, and the LBP texture feature matrix database was maintained. This feature matrix database had 12 matrices per 110 subjects. In addition to this, two more sample databases were maintained with 10 and 8 feature matrices per subject. These three sample feature matrix databases were used to train and test the SVM classification model. The classification accuracies and elapsed time are presented in Table 4.

During the training of the SVM model, different variations were tried for feature selection, and different numbers of best features were used for classification. Based on observations, it was decided to use forward sequential feature selection, and the five best features were selected to train the SVM classification model for all three image databases. 

### 4.2. Finger Vein Biometric

In this step, image classification was performed for the databases. The preprocessed images were fed into the pre-trained convolutional neural networks for transfer learning. The MATLAB code was executed on a laptop with Intel Core i7, 11th generation microprocessor—2.80 GHz supported with GPU NVIDIA GeForce MX450. 

For all three databases, the database was divided into training and testing images with ratios of 0.8 and 0.2, respectively. MATLAB 2021a’s ‘splitEachLabel’ function was used for the division of the databases into training and test images. The training of the CNN model was performed using the ‘trainNetwork’ function. The hyperparameters for CNN models are mentioned here. Training was performed using stochastic gradient descent with momentum. An initial learning rate of 0.001 and a mini-batch size of 64 images were selected. Training was performed for eight epochs. To avoid model overfitting, data augmentation/transformation, a reduced mini-batch size and an optimized number of epochs were employed. 

There were two approaches used to experiment with the databases:

- In Approach 1, the image intensity optimization step is skipped while preprocessing the images. The observed performance parameters are in Table 5.
- In Approach 2, the image intensity optimization step is included in the preprocessing of the images. The observed performance parameters are in Table 5.

#### 4.2.1. Approach 1

1. NIRHI Database:

For the proposed database, 95.29%, 93.40% and 91.13% identification accuracies were achieved through transfer learning through AlexNet, VGG16 and VGG19, respectively.

2. HKPU Database:

For the HKPU database, 89.68%, 92.53% and 90.04% identification accuracies were observed through the transfer learning of AlexNet, VGG16 and VGG19 respectively.

3. UTFVP Database:

For the UTFVP database, 83.79%, 82.25% and 85.08% identification accuracies were attained for the transfer learning of AlexNet, VGG16 and VGG19, respectively.

#### 4.2.2. Approach 2

1. NIRHI Database:

For the proposed database, 96.96%, 96.57%, and 95.86% identification accuracies were achieved through transfer learning through AlexNet, VGG16 and VGG19, respectively.

2. HKPU Database:

For the HKPU database, 89.68%, 92.53% and 90.04% identification accuracies were observed through the transfer learning of AlexNet, VGG16 and VGG19, respectively.

3. UTFVP Database:

For the UTFVP database, 83.79%, 82.25% and 85.08% identification accuracies were attained for the transfer learning of AlexNet, VGG16 and VGG19 respectively.

### 4.3. Evaluation Metrics

For both biometric modalities, i.e., Finger Textures and Finger Veins, the models were trained, and different evaluation metrics were calculated, which included Precision, Recall/Sensitivity (True Positive Rate), Specificity (True Negative Rate) and False Positive Rate.

The ROC curves were drawn and AUC values were calculated for the curves (False Positive Rate vs. True Positive Rate) for some Support Vector Machine and AlexNet and VGG16 Convolutional Neural Network models. The detailed discussion is reported in the Simulation and Discussion section.

### 4.4. Fuzzy Inference System

**Figure 8:**
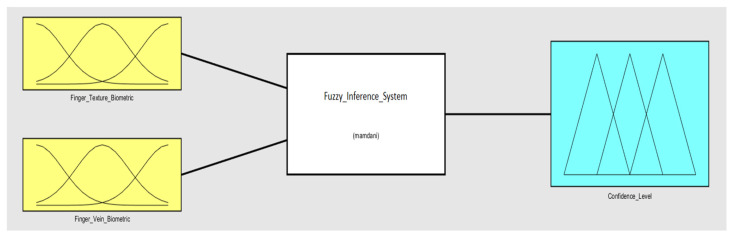
Fuzzy Rule-Based Inference System.

The individual identification results of the two modalities of concern were fused by employing a Fuzzy Rule-Based Inference system, as shown in Figure 8. The fuzzy inference system gives confidence level output in the range of 0–1.

There are three steps in designing the Fuzzy Inference System: 1. fuzzification (assigning linguistic variables to the real values of inputs and selecting suitable membership functions), 2. inference (defining the fuzzy rules) and 3. defuzzification (making the fuzzified result understandable in the real world). Each step is discussed below one by one.

#### 4.4.1. Fuzzification of the Finger Texture Biometric

**Figure 9:**
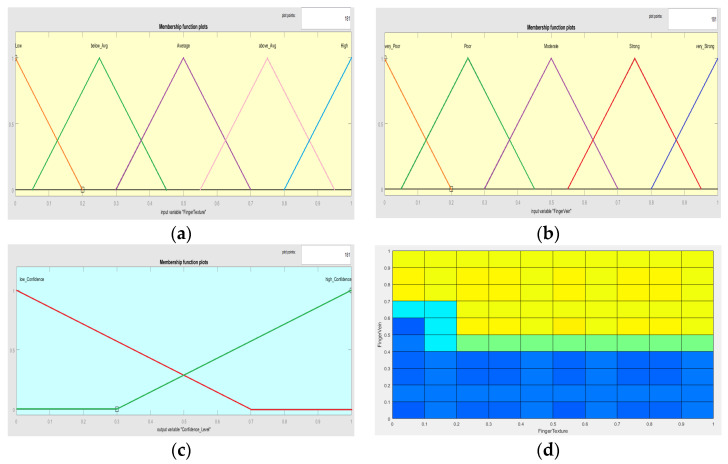
Fuzzification of input and output variables. (**a**) Finger Texture biometric. (**b**) Finger Vein biometric. (**c**) Confidence level. (**d**) Surface for Fuzzy Inference System.

In the first step, real-world values are to converted into fuzzified linguistic variables. Five linguistic variables were used for the Finger Texture biometric, i.e., Low, below_Avg, Average, above_Avg and High. According to the best-observed behavior of the Fuzzy Inference System, triangular membership functions were assigned to all five linguistic variables, as shown in Figure 9a.

#### 4.4.2. Fuzzification of the Finger Vein Biometric

The same procedure as the first step for the Finger Vein biometric was repeated, and real-world values were converted into fuzzified linguistic variables. Five linguistic variables were defined for the individual confidence score of the Finger Vein biometric, i.e., very_Poor, Poor, Moderate, Strong and very_Strong. According to the best-observed behavior of the Fuzzy Inference System, triangular membership functions were assigned to all five linguistic variables, as shown in Figure 9b.

#### 4.4.3. Fuzzification of the Output Confidence Score

For the output of the Fuzzy Inference System, two linguistic variables were defined, i.e., low_Confidence and high_Confidence. According to the best-observed behavior of the Fuzzy Inference System, triangular membership functions were selected for these two linguistic variables, as shown in Figure 9c.

#### 4.4.4. Inference—Fuzzy Rules

In the second step, a total of 25 fuzzy rules were defined and implemented to take advantage of the multimodal biometric system. A total of 5 rules out of 25 are given here as an example.

“input1==Low and input2== Moderate => output1=low_Confidence (1)”;
“input1== below_Avg and input2== Moderate => output1=low_Confidence (1)”;
“input1==Average and input2== Moderate => output1=high_Confidence (1)”;
“input1==above_Avg and input2== Moderate => output1=high_Confidence (1)”;
“input1==High and input2== Moderate => output1=high_Confidence (1)”.

The details of the system are given in Table 6. According to the individual reliability of the identification results, the Finger Vein biometric was treated as a strong candidate in comparison with the Finger Texture biometric. For example, if the Finger Vein biometric system identified a candidate subject as a member of subject class-1 with the membership function of Moderate, and if the Finger Texture biometric system identifies a candidate subject as a member of subject class-2 with the membership function of Average, the decision of the prior would be considered true. On the other hand, if the Finger Vein biometric system identified a candidate subject as a member of subject class-3 with the membership function of Poor, and if the Finger Texture biometric system identified a candidate subject as a member of subject class-2 with the membership function of High, the decision of the latter would be considered true.

The surface of the Fuzzy Inference System according to the defined rules is shown in Figure 9d.

#### 4.4.5. Defuzzification

The Mamdani fuzzy rule-based system was employed for the fusion of individual results of biometric modalities; hence, for the defuzzification of the final result of identification, the centroid method was chosen. In this method, the effect of fuzzified input membership functions is mapped on the output variable’s membership functions plot. Then, the centroid of the area under the mapped portion of the output membership function graph is calculated, which represents the final real-world value for the identification confidence score. This score is in the range of 0–1. 

**Figure 10:**
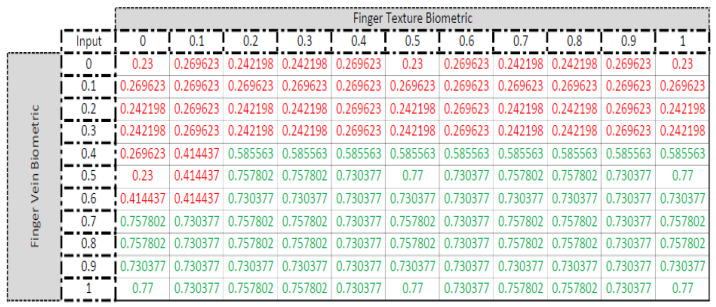
Output confidence scores against individual confidence scores as inputs for the Fuzzy Inference System. Red colour—Low confidence, Green colour—High confidence.

If the output of the Fuzzy Inference System is low, indicated with a red font color in Figure 10, this means that the candidate subject does not belong to that particular class. Otherwise, if the output of the Fuzzy Inference System is high, indicated with green font color in Figure 10, then the candidate subject is declared a member of that particular subject class according to the individual identification results of individual biometric modalities.

### 4.5. Capturing Training and Testing Times

The experimental setup included the use of a camera to note down the training and testing times for the models. Whenever a model was trained or tested, the power settings for the used laptop were set to never go into sleep mode, and the camera remained ON to capture the video of the laptop’s monitor screen. In this way, the training and testing times for the employed models were observed. 

## 5. Simulation and Discussion

**Figure 11:**
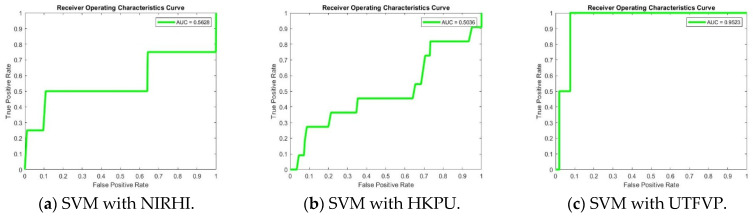

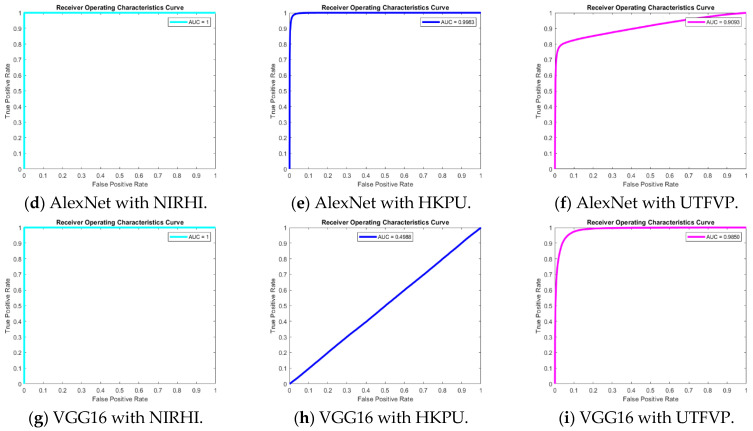
Sample ROC curves for Finger Texture (**a**–**c**) and Finger Vein (**d**–**i**) biometrics.

Figure 11 has some observed Receiver Operating Characteristics curves for the Finger Texture and Finger Vein biometric algorithm models. The area under the curve (AUC) is also shown for each of the ROC curves. These graphs show that the Finger Vein algorithm models performed better in comparison with the Finger Texture algorithm models, as the AUC values explain. According to the theory of performance parameters for a machine learning system, a False Positive Rate is called a False Rejection Rate (FRR), and a False Negative Rate is called a False Acceptance Rate (FAR). Higher values of area under the curve show better performance for an identification model. For the calculation of FRR and FAR for different identification thresholds, we employed MATLAB functions and plotted ROC curves.

### 5.1. Finger Texture Biometric

The observations listed in Table 3 and Table 4 explain that the values for FRR are smaller than the values for FAR for all three databases. From this, it may be deduced that the False Positive Rate values are much lower for the NIRHI database in comparison with the HKPU and the UTFVP databases. In addition to this, it is evident that the overall accuracy values are good for NIRHI, and a bigger sample size was selected. It may also be concluded easily that the overall accuracy values are better for UTFVP in comparison with HKPU, but NIRHI outperformed both the other databases. Figure 11a–c shows some sample ROC curves, in which the False Positive Rate is plotted against the True Positive Rate. It may be observed that the AUC values are better for the UTFVP database than the NIRHI and HKPU databases, but the overall accuracy is better for NIRHI in comparison with the other two databases, as listed in Table 2 and Table 3.

### 5.2. Finger Vein Biometric

The observations listed in Table 4 are related to the Finger Vein biometric module. It is evident that the FRR values are smaller than the values for FAR in all approaches. The overall accuracy values are better for Approach 2 in comparison to Approach 1. In addition to this, it may also be observed that NIRHI outperformed HKPU and UTFVP in the case of the overall performance accuracy, precision and recall values.

Further, HKPU performed better than UTFVP for all three pre-trained CNNs used (AlexNet, VGG1, and VGG19). The ROC curves, plotted for the False Positive Rate vs. True Positive Rate, shown in Figure 11d–i, demonstrate that the AUC is higher for the combined database comprising all three fingers, i.e., the center, ring and index fingers, for NIRHI in comparison with the other two databases, i.e., HKPU and UTFVP.

### 5.3. Fuzzified Multimodal Biometric

Figure 10 shows the output of the Fuzzy Inference System in grid form. The confidence values for individual biometric modalities from 0 to 1 were applied as input, and the observed output is listed. A green font color is used for output confidence values above 0.5, whereas below 0.5, output confidence values have a red font color. It is evident that, when the Finger Vein confidence value was above 0.6 for some candidate image to be classified as a member of a particular subject class, the decision of the Finger Texture confidence value did not affect the overall identification result. On the other hand, if the confidence value for the Finger Vein biometric fell below 0.3 for a candidate image to be classified as not a member of a particular subject class, then the Finger Texture confidence value did not affect the overall identification result.

The experimental results shown in Table 7 show that the NIRHI database outperformed the HKPU and UTFVP databases in identification accuracy and other performance metrics of individual modalities, as well as for the overall accuracy and other performance metrics of the multimodal biometric identification system. The experiments were performed with and without applying the image intensity regularization step for the Finger Vein biometric only. The Finger Texture biometric algorithm was applied to the original databases. For both methodologies, the proposed database had better identification accuracies in comparison with the two acquired databases. Table 4 also clearly reports that the defined fuzzy rules supported the results of individual modalities, and an enhanced overall identification accuracy was achieved after fusion. We also note that the fuzzy rules defined for AlexNet remained valid for VGG16 as well as VGG19.

#### 5.3.1. Training Time vs. Accuracy

**Figure 12:**
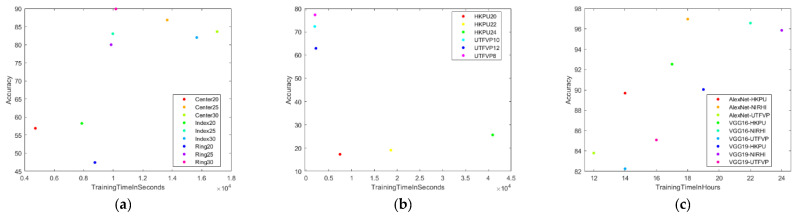
Training time versus accuracy scatter plots. (**a**) SVM—NIRHI. (**b**) SVM—HKPU and UTFVP. (**c**) CNN—all databases.

Figure 12 shows the training time versus accuracy scatter plots for different Finger Texture and Finger Vein models. The training time was much lower for most of the SVM models, and the accuracy was higher for NIRHI compared to the HKPU and UTFVP databases. It is also obvious that the performance of AlexNet was better than VGG16 and VGG19, with a higher accuracy and lower training time for the Finger Vein biometric employing all three databases.

#### 5.3.2. Testing Time vs. Accuracy

**Figure 13:**
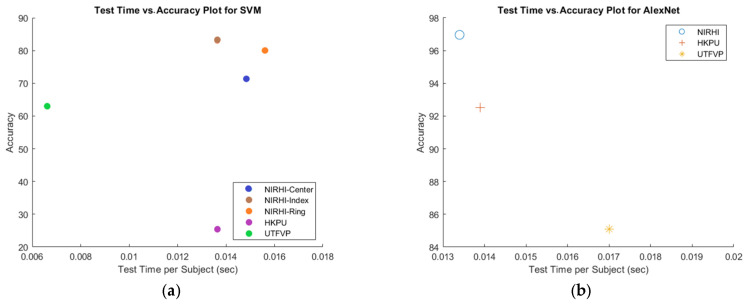
Testing time vs. accuracy scatter plots. (**a**) Testing candidate image time vs. accuracy for SVM. (**b**) Testing candidate image time vs. accuracy for CNN.

Figure 13 shows the testing time versus accuracy scatter plots for the AlexNet and SVM models. Figure 13b shows that accuracy was the highest and testing time was the lowest for the NIRHI database in comparison with the other two databases for AlexNet. From Figure 13a, it is evident that, for SVM, NIRHI had the highest accuracy values, and the testing time for candidate images was also the highest. However, comparatively, the testing time for NIRHI was not much higher (in milliseconds). Hence, it may perform satisfactorily in our proposed multimodal biometric system without creating any extra delay in making an identification decision.

## 6. Comparative Analysis

### 6.1. Accuracy

As listed in Table 2, Table 3 and Table 4, the best accuracy for the Finger Texture biometric was 89.892% for NIRHI, whereas for HKPU and UTFVP, the best accuracy values were 25.634% and 77.273%, respectively. Further, the Finger Vein biometric best accuracy values also evidence that NIRHI outperformed the other two databases, i.e., HKPU and UTFVP, for both Approaches 1 and 2. After applying the Fuzzy Inference Fusion System at the confidence score level, the overall accuracy values for the multimodal biometric system were 99.62%, 92.95% and 86.32% for NIRHI, HKPU and UTFVP, respectively.

In comparison with current state-of-the-art systems, the proposed system’s performance was better in terms of overall accuracy. Table 7 shows that a fuzzified overall identification accuracy of 99.62% was achieved for the proposed multimodal system for the NIRHI database. The authors of [26] reported an overall recognition accuracy of 99.51% for their fingerprint- and Finger-Vein-based multimodal biometric system. The authors of [28] reported an overall identification accuracy of 99.5%. They employed Finger Vein and palm vein biometrics for the identification of individuals in their research work. Further, other researchers [29] reported an overall identification accuracy of 99.33% for their proposed multimodal biometric system. They proposed an identification system based on iris, palm vein and Finger Vein biometrics. Comparisons of accuracy and other metrics with current state-of-the-art systems are listed in Table 8.

### 6.2. Equal Error Rate

**Figure 14:**
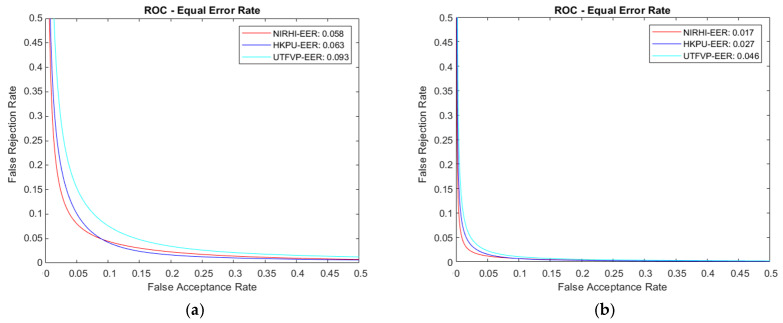
Equal Error Rate ROC curve for the multimodal system using NIRHI. (**a**) Approach 1 for AlexNet. (**b**) Approach 2 for AlexNet.

The Equal Error Rate (ERR) is a statistical performance metric that indicates at which point on the ROC curve the value of the False Acceptance Rate (FAR) is equal to the False Rejection Rate (FRR). A lower value for the EER is desirable for better identification performance of a biometric system. EER values for all three databases are shown for Approaches 1 and 2 in the ROC curves of Figure 14a and Figure 14b, respectively.

Table 9 shows a comparison of EER values for the proposed system using AlexNet with research articles [19,20]. According to the listed data, the best EER of 0.058 and 0.017 were achieved for Approaches 1 and 2 when working with the NIRHI database. The best three EER values reported in the cited research articles are listed in the table. Hence, the proposed system demonstrates a better identification performance.

## 7. Conclusions

Finally, according to the details discussed in previous sections, the following conclusions may be extracted:

1. The intensity optimization scheme positively affected the overall accuracy value. Hence, better accuracies were achieved for Approach 2 in comparison with Approach 1.
2. Finger Texture features were extracted from NIR images in this research work. These extracted features are intrinsic and hence less vulnerable to the skin conditions of the subjects.
3. The listed observed performance parameters in Table 3 show that the optimal sample size is 25 images per subject to achieve high accuracy values above 80% while having an optimal model training time. If the sample size is increased to 30 images per subject, there is a small variation in the achieved accuracy along with increases in the model training time. Therefore, 25 images per subject is an optimal sample size for the NIRHI database for the Finger Texture algorithm.
4. It may be deduced from Table 4 that sample sizes of 24 images and 10 images per subject are optimal sample sizes for the HKPU and UTFVP databases, respectively, for the Finger Texture algorithm.
5. A multimodal biometric system is proposed with a demonstrated high identification accuracy of 99.62% for the NIRHI database.
6. The Fuzzy Rule-Based Inference system generated enhanced accuracy values for the multimodal system in comparison with the unimodal accuracies.
7. It is concluded that the Finger Texture biometric algorithm results for the NIRHI database are better in terms of evaluation metrics compared to the results for the HKPU and UTFVP databases.
8. Further, it is concluded that, for the Finger Vein biometric algorithm, AlexNet performed better in terms of evaluation metrics in comparison with VGG16 and VGG19 for Approaches 1 and 2.
9. In addition to this, after comparative analysis, it is also concluded that NIRHI is a database that is capable of training and testing a biometric system based on Near-Infra-Red hand/finger images.
10. It is also concluded that, due to optimal testing times, the proposed system may be used in real-time applications. Hence, real-life implementation is possible for the proposed system.

The proposed system has the following limitations:

1. The proposed system depends upon biometric modalities related to the human hand and fingers. A human subject that has some disability or injury related to hands or fingers will not be able to use this system.
2. An NIR camera or camera with mounted NIR filters is expensive and may not be arranged without enough allocated funding. Therefore, it is an expensive system overall.
3. Computers or laptops with installed graphical processing units are required for the training and testing of the CNN and SVM models. Therefore, high-performance computational resources are also required, which are also expensive.

In the future, the following may be the research directions after this research work:

- As the proposed system employs NIR images, and due to the intrinsic nature of the features present in the input images, the capability to resist any imposter attack is enhanced exponentially. In the future, the proposed system may be evaluated for its capability to observe and quantify different imposter attacks.
- Some medical issues generally found in elderly subjects, like vein thrombosis and connective tissue diseases, may affect Finger Vein patterns. Moreover, during a period, registered subjects may have some injuries resulting in damage or changes in Finger Vein and Finger Texture features. This change in biometric features may increase the False Rejection Rate and decrease reliability. This behavior of change in Finger Vein structure may be observed, and some predictions and remedies may be suggested in future work.
- Performance variations in the proposed system due to environmental variability (like changes in room temperature, etc.) and subject diversity (like gender, age factor, etc.) may be observed in future research work.

## Figures and Tables

**Table 1:** Summary of cited state-of-the-art multimodal biometric systems.

Cited Articles	Modalities Employed	Methodology and Shortcomings	Reported Evaluation Metrics and ROC Curves
[3]	1. Finger Vein 2. Hand Geometry 3. Pulse Response	CNN model training and testing for Finger Veins, Handcrafted technique for Hand Geometry, Fuzzy Fusion for the final result, EER was not reported.	Accuracy, Precision, Recall, FAR vs. GAR, Threshold vs. FAR/FRR
[17]	1. Finger Vein 2. Fingerprints	Feature-level fusion, LBP for feature extraction and SVM as the classifier, Accuracy, FRR, Precision and Recall were not reported. May only handle small datasets.	FAR, Recognition Rate
[20]	1. Finger Vein 2. Fingerprint	CNN models, score-level fusion for identification results, Accuracy, Precision and Recall were not reported.	FAR vs. GAR, EER
[21]	1. Iris 2. Face 3. Finger Vein	CNN models, feature- and score-level fusion, FAR, FRR, EER, training and testing times were not reported.	Accuracy, Rank vs. Recognition Rate
[26]	1. Finger Vein 2. Fingerprint 3. Face	Random forest classifier, softmax function and CNN were used for Finger Veins, faces and fingerprints, score-level fusion was implemented. No other metric was reported except Accuracy.	Accuracy
[28]	1. Finger Vein 2. Palm Vein	2D Gabor Filter, Gradient Filter was used for feature extraction, Fuzzy score-level fusion was used. Precision, Recall, etc., were not reported.	Accuracy, Threshold vs. FRR, FRR vs. FAR, EER
[29]	1. Iris 2. Palm Vein 3. Finger Vein	The best from the Gabor Filter, LBP, LDA, PCA was used for feature extraction, feature-level, decision-level and hybrid fusion were proposed. Precision, Recall, etc., were not reported.	Accuracy, Recognition Rate graphs were reported

**Table 2:** Confusion matrix.

Actual ↓\Predicted →	Positive	Negative
True	True Positive (TP)	True Negative (TN)
False	False Positive (FP)	False Negative (FN)

**Table 3:** Performance parameters for NIRHI database.

Sample Size	Performance Parameter	Center Finger	Index Finger	Ring Finger
30	Accuracy	83.6036	81.9820	89.8919
	Model Training Time (s)	17,033.4894	15,657.2421	10,176.6519
	Function Evaluation Time (s)	387.2414	335.1072	754.9668
	Precision	0.8331	0.8227	0.8156
	Recall (True Positive Rate)	0.8232	0.8163	0.8076
	F1 Score	0.8281	0.8195	0.8116
	True Negative Rate	0.9990	0.9990	0.9990
	False Positive Rate	9.6088 × 10^−4^	9.9270 × 10^−4^	0.0010
	False Negative Rate	0.1768	0.1827	0.1921
25	Accuracy	86.8108	83.0270	80
	Model Training Time (s)	13,647.683	9973.0583	9855.4196
	Function Evaluation Time (s)	294.6148	312.2454	367.9968
	Precision	0.8625	0.8304	0.8217
	Recall (True Positive Rate)	0.8576	0.8209	0.8116
	F1 Score	0.8600	0.8256	0.8166
	True Negative Rate	0.9992	0.9990	0.9990
	False Positive Rate	7.7409 × 10^−4^	9.6651 × 10^−4^	0.0010
	False Negative Rate	0.1424	0.1778	0.1868
20	Accuracy	56.8919	58.2432	47.4324
	Model Training Time (s)	4727.9013	7874.1529	8758.2269
	Function Evaluation Time (s)	166.4988	270.3589	303.5633
	Precision	0.6409	0.6230	0.4618
	Recall (True Positive Rate)	0.6146	0.5899	0.4424
	F1 Score	0.6275	0.606	0.4519
	True-Negative Rate	0.9974	0.9972	0.9962
	False Positive Rate	0.0026	0.0028	0.0038
	False Negative Rate	0.3804	0.4084	0.5547

**Table 4:** Performance parameters for HKPU and UTFVP databases.

Sample Size (HKPU)	Performance Parameter	Evaluation Metrics	Sample Size (UTFVP)	Performance Parameter	Evaluation Metrics
24	Accuracy	25.6342	12	Accuracy	62.8788
	Model Training Time (s)	40,930.4645		Model Training Time (s)	2170.757
	Function Evaluation Time (s)	3583.3136		Function Evaluation Time (s)	96.9479
	Precision	0.2672		Precision	0.5952
	Recall (True Positive Rate)	0.2737		Recall (True Positive Rate)	0.5778
	F1 Score	0.2704		F1 Score	0.5864
	True Negative Rate	0.9953		True Negative Rate	0.9962
	False Positive Rate	0.0047		False Positive Rate	0.0038
	False Negative Rate	0.7226		False Negative Rate	0.4148
22	Accuracy	19.0962	10	Accuracy	72.2727
	Model Training Time (s)	18,611.9825		Model Training Time (s)	1917.2405
	Function Evaluation Time (s)	2121.2213		Function Evaluation Time (s)	83.7306
	Precision	0.1523		Precision	0.6581
	Recall (True Positive Rate)	0.1784		Recall (True Positive Rate)	0.6461
	F1 Score	0.1643		F1 Score	0.6520
	True Negative Rate	0.9947		True Negative Rate	0.9969
	False Positive Rate	0.0053		False Positive Rate	0.0031
	False Negative Rate	0.8230		False Negative Rate	0.3420
20	Accuracy	17.3077	8	Accuracy	77.2727
	Model Training Time (s)	7458.5968		Model Training Time (s)	1971.2457
	Function Evaluation Time (s)	720.7739		Function Evaluation Time (s)	104.3346
	Precision	0.1480		Precision	0.7691
	Recall (True Positive Rate)	0.1623		Recall (True Positive Rate)	0.7588
	F1 Score	0.1548		F1 Score	0.7639
	True Negative Rate	0.9946		True Negative Rate	0.9980
	False Positive Rate	0.0054		False Positive Rate	0.0020
	False Negative Rate	0.8349		False Negative Rate	0.2145

**Table 5:** Performance parameters for Finger Vein algorithm.

Databases	Performance Parameters	Approach 1			Approach 2		
		AlexNet	VGG16	VGG19	AlexNet	VGG16	VGG19
NIRHI	Accuracy	95.29%	93.40%	91.13%	96.96%	96.57%	95.86%
	Precision	95.29%	93.40%	91.13%	96.95%	96.57%	95.86%
	Recall	95.29%	93.40%	91.13%	96.95%	96.57%	95.86%
	False Positive Rate	4.59 × 10^−4^	8.90 × 10^−3^	5.60 × 10^−3^	2.75 × 10^−6^	2.34 × 10^−5^	6.3 × 10^−4^
	True Negative Rate	99.75%	99.49%	99.17%	100%	100%	100%
	False Negative Rate	1.56 × 10^−3^	0.0012	0.0026	5.07 × 10^−4^	0.0043	0.0065
HKPU	Accuracy	81.93%	89.01%	87.65%	89.68%	92.53%	90.04%
	Precision	81.93%	89.01%	87.65%	89.68%	92.53%	90.04%
	Recall	81.93%	89.01%	87.65%	89.68%	92.53%	90.04%
	False Positive Rate	0.0012	7.09 × 10^−4^	3.57 × 10^−3^	0.0021	3.92 × 10^−3^	5.09 × 10^−3^
	True Negative Rate	99.88%	99.93%	99.95%	98.87%	99.27%	99.32%
	False Negative Rate	0.1807	0.1099	0.134	0.0561	0.0086	0.0103
UTFVP	Accuracy	78.79%	79.12%	80.70%	83.79%	82.25%	85.08%
	Precision	79.52%	79.12%	80.70%	83.79%	82.25%	85.08%
	Recall	80.12%	79.12%	80.70%	83.79%	82.25%	85.08%
	False Positive Rate	0.0017	0.0019	0.0022	0.0025	0.0029	0.0034
	True Negative Rate	99.83%	99.81%	99.79%	98.56%	98.14%	98.02%
	False Negative Rate	0.1988	0.2088	0.201	0.1568	0.182	0.1692

**Table 6:** Details for Fuzzy Rule-Based Inference System.

Basic Definitions	Inputs and Output	Rules
[System] Name=‘FIS_2′ Type=‘mamdani’ Version=2.0 NumInputs=2 NumOutputs=1 NumRules=25 AndMethod=‘min’ OrMethod=‘max’ ImpMethod=‘min’ AggMethod=‘max’ DefuzzMethod=‘centroid’	[Input1] Name=‘FingerTexture’ Range=[0 1] NumMFs=5 MF1=‘Low’:’trimf’,[−0.2 0 0.2] MF2=‘below_Avg’:’trimf’,[0.05 0.25 0.45] MF3=‘Average’:’trimf’,[0.3 0.5 0.7] MF4=‘High’:’trimf’,[0.8 1 1.2] MF5=‘above_Avg’:’trimf’,[0.55 0.75 0.95] [Input2] Name=‘FingerVein’ Range=[0 1] NumMFs=5 MF1=‘very_Poor’:’trimf’,[−0.2 0 0.2] MF2=‘Poor’:’trimf’,[0.05 0.25 0.45] MF3=‘Moderate’:’trimf’,[0.3 0.5 0.7] MF4=‘very_Strong’:’trimf’,[0.8 1 1.2] MF5=‘Strong’:’trimf’,[0.55 0.75 0.95] [Output1] Name=‘Confidence_Level’ Range=[0 1] NumMFs=2 MF1=‘high_Confidence’:’trimf’,[0.3 1 1.6] MF2=‘low_Confidence’:’trimf’,[−0.7 0 0.7]	[Rules] 1 1, 2 (1) : 1 1 2, 2 (1) : 1 1 3, 2 (1) : 1 1 4, 1 (1) : 1 1 5, 1 (1) : 1 2 1, 2 (1) : 1 2 2, 2 (1) : 1 2 3, 1 (1) : 1 2 4, 1 (1) : 1 2 5, 1 (1) : 1 3 1, 2 (1) : 1 3 2, 2 (1) : 1 3 3, 1 (1) : 1 3 4, 1 (1) : 1 3 5, 1 (1) : 1 4 1, 2 (1) : 1 4 2, 2 (1) : 1 4 3, 1 (1) : 1 4 4, 1 (1) : 1 4 5, 1 (1) : 1 5 1, 2 (1) : 1 5 2, 2 (1) : 1 5 3, 1 (1) : 1 5 4, 1 (1) : 1 5 5, 1 (1) : 1

**Table 7:** Individual and fused multimodal observed Accuracy, Precision and Recall.

Modality	Accuracy			Precision			Recall		
	NIRHI	HKPU	UTFVP	NIRHI	HKPU	UTFVP	NIRHI	HKPU	UTFVP
Finger Texture	86.81%	25.63%	77.27%	86.25%	26.71%	76.91%	85.76%	27.37%	75.88%
Finger Vein	96.96%	92.53%	85.08%	96.95%	92.53%	85.08%	96.95%	92.53%	85.08%
Multimodal	99.62%	92.95%	86.32%	98.12%	91.30%	83.86%	99.47%	91.49%	83.93%

**Table 8:** Accuracy comparison with currently proposed best systems.

Methodology	Accuracy	Precision	Recall	F1 Score
Proposed in [26]	99.51%	-	-	-
Proposed in [28]	99.50%	-	-	-
Proposed in [29]	99.33%	-	-	-
Proposed in [30]	99.00%	97.00%	100%	98%
Proposed in this article	99.62%	98.12%	99.47%	99.60%

**Table 9:** Equal Error Rate comparison with currently proposed best systems.

EER VALUES →	Proposed AlexNet		Cited Research Article	
↓ DATABASE	Approach 1	Approach 2	[19]	[20]
NIRHI	0.058	0.017	0.019	0.7859
HKPU	0.063	0.027	0.0243	0.8255
UTFVP	0.093	0.046	0.0338	0.8265

## Data Availability

NIRHI database is available on request. The format of requisition form may be found in [3].
